# Supplementary material for: Studying mixed-species biofilms of Candida albicans and Staphylococcus aureus using evolutionary game theory
Source: PLoS One. 2024 Mar 6;19(3):e0297307. doi: 10.1371/journal.pone.0297307 (PMC10917284; doi:10.1371/journal.pone.0297307)
Supplement: S1 File — (PDF) [file pone.0297307.s001.pdf]

Studying mixed-species biofilms of *Candida albicans* and *Staphylococcus aureus* using Evolutionary Game Theory

- Supporting Information 2 -

Sybille Dühring[1*], Stefan Schuster[1],

1 Dept. of Bioinformatics, Friedrich-Schiller-University Jena, Jena, Germany

* sybille.duehring@uni-jena.de

Stability analysis of the five fixed points of the game

In our study, we derived the following replicator equation system for our game

$$\begin{aligned}\dot{x} =& x(1-x)(E_Y - rE_H + I_2 + 2f_{ar} - y((1-b)I_1 + I_2 + f_{ar})) \quad \text{and} \\ \dot{y} =& y(1-y)(bI_1 - I_2 + f_2 + x(I_2 - bI_1)),\end{aligned} \tag{1}$$

with four trivial fixed points $(x^*, y^*) \in \{0, 1\}$ and for $(x^*, y^*) \in (0, 1)$ an inner fixed point

$$(\hat{x}, \hat{y}) = \left(\frac{bI_1 - I_2 + f_2}{bI_1 - I_2}, \frac{E_Y - rE_H + I_2 + 2f_{ar}}{I_2 + (1-b)I_1 + f_{ar}} \right). \tag{2}$$

The inner fixed point $(\hat{x}, \hat{y})$ only exists provided that

$$\begin{aligned}&0 < \frac{bI_1 - I_2 + f_2}{bI_1 - I_2} < 1 \quad \text{and} \\ &0 < E_Y - rE_H + I_2 + 2f_{ar} < I_2 + (1-b)I_1 + f_{ar}.\end{aligned} \tag{3}$$

To analyse the stability of the fixed points we use linearisation. We determine the Jacobian matrix J of our replicator equation system:

$$J = \begin{pmatrix} (1-2x)(I_2 + E_Y + 2f_{ar} - rE_H - y(\sigma_1)) & -x(1-x)(\sigma_1) \\ y(1-y)(\sigma_2) & (1-2y)(-\sigma_2 + f_2 + x(\sigma_2)) \end{pmatrix} \tag{4}$$

with σ_1 and σ_2 being

$$\begin{aligned}\sigma_1 =& I_2 + f_{ar} + (1-b)I_1 \text{ and} \\ \sigma_2 =& I_2 - bI_1.\end{aligned} \tag{5}$$

For a fixed point (x^*, y^*) to be asymptotically stable the eigenvalues λ_1 and λ_2 of the Jacobian matrix $J_{(x^*,y^*)}$ at the point (x^*, y^*) have to be negative. For the four trivial fixed points $(x^*, y^*) \in \{0, 1\}$ the eigenvalues λ_1 and λ_2 of $J_{(x^*,y^*)}$ lie on the main diagonal of $J_{(x^*,y^*)}$.

Fixed point $(0,0)$

For $(x^*, y^*) = (0,0)$ we find

$$J_{(0,0)} = \begin{pmatrix} I_2 + E_Y + 2f_{ar} - rE_H & 0 \\ 0 & bI_1 - I_2 + f_2 \end{pmatrix}. \tag{6}$$

Hence for $(x^*, y^*) = (0,0)$ to be asymptotically stable the two stability conditions

$$\begin{aligned} rE_H - E_Y &> I_2 + 2f_{ar} \quad \text{and} \\ f_2 &< I_2 - bI_1 \end{aligned} \tag{7}$$

need to hold true.

Fixed point $(1,0)$

For $(x^*, y^*) = (1,0)$ we find

$$J_{(1,0)} = \begin{pmatrix} rE_H - E_Y - 2f_{ar} - I_2 & 0 \\ 0 & f_2 \end{pmatrix}. \tag{8}$$

For $(x^*, y^*) = (1,0)$ to be asymptotically stable the two stability conditions

$$\begin{aligned} rE_H - E_Y &< I_2 + 2f_{ar} \quad \text{and} \\ f_2 &< 0 \end{aligned} \tag{9}$$

need to hold true.

Fixed point $(0,1)$

For $(x^*, y^*) = (0,1)$ we find

$$J_{(0,1)} = \begin{pmatrix} E_Y + f_{ar} - rE_H - (1-b)I_1 & 0 \\ 0 & I_2 - f_2 - bI_1 \end{pmatrix}. \tag{10}$$

For $(x^*, y^*) = (0,1)$ to be asymptotically stable the two stability conditions

$$\begin{aligned} E_Y - rE_H &< (1-b)I_1 - f_{ar} \quad \text{and} \\ f_2 &> I_2 - bI_1 \end{aligned} \tag{11}$$

need to hold true.

Fixed point $(1,1)$

For $(x^*, y^*) = (1,1)$ we find

$$J_{(1,1)} = \begin{pmatrix} rE_H - f_{ar} - E_Y + (1-b)I_1 & 0 \\ 0 & -f_2 \end{pmatrix}. \tag{12}$$

Hence, for $(x^*, y^*) = (1,1)$ to be asymptotically stable the two stability conditions

$$\begin{aligned} E_Y - rE_H &> (1-b)I_1 - f_{ar} \quad \text{and} \\ f_2 &> 0 \end{aligned} \tag{13}$$

need to hold true.

Fixed point $(\hat{x}, \hat{y})$

For the inner fixed point $(\hat{x}, \hat{y})$ we find

$$J_{(\hat{x},\hat{y})} = \begin{pmatrix} 0 & \sigma_3 \\ -\sigma_4 & 0 \end{pmatrix} \tag{14}$$

with σ_3 and σ_4 being

$$\begin{aligned} \sigma_3 &= \frac{(f_2(f_2 - I_2 + bI_1)((1-b)I_1 + I_2 + f_{ar}))}{(I_2 - bI_1)^2} \quad \text{and} \\ \sigma_4 &= \frac{((I_2 - bI_1)(I_2 + E_Y + 2f_{ar} - rE_H)(E_Y + (b-1)I_1 + f_{ar} - rE_H))}{((1-b)I_1 + I_2 + f_{ar})^2}. \end{aligned} \tag{15}$$

From this, we derive

$$\lambda_{1,2} = \pm\sqrt{\frac{\sigma_5}{(bI_1 - I_2)((1-b)I_1 + I_2 + f_{ar})}} \tag{16}$$

with σ_5 being

$$\sigma_5 = f_2(I_2 - f_2 - bI_1)(I_2 + E_Y + 2f_{ar} - rE_H)((1-b)I_1 - E_Y - f_{ar} + rE_H). \tag{17}$$

If the term under the square root is positive the fixed point $(\hat{x}, \hat{y})$ is unstable. In case of a negative term under the square root, we find complex conjugated eigenvalues with real part equals 0, indicating a centre or spiral. By definition $(1-b)I_1$ and I_2 are positive. In case of artificially added farnesol as a medical adjuvant $f_{ar} > 0$, otherwise 0. Therefore

$$(1-b)I_1 + I_2 + f_{ar} > 0. \tag{18}$$

From the existence condition of $(\hat{x}, \hat{y})$ (Eq 3) we know that

$$0 < E_Y - rE_H + I_2 + 2f_{ar} \tag{19}$$

and further derive that

$$E_Y - rE_H + f_{ar} < (1-b)I_1 \tag{20}$$

and with that

$$0 < (1-b)I_1 - E_Y - f_{ar} + rE_H. \tag{21}$$

Hence, for the term under the square root to be negative the following equation must hold true:

$$\frac{f_2(I_2 - f_2 - bI_1)}{(bI_1 - I_2)} < 0. \tag{22}$$

For $f_2 < 0$ we find that Eq 22 holds true for $\frac{(I_2 - f_2 - bI_1)}{(bI_1 - I_2)} > 0$. However, this is a contradiction to the existence condition (Eq 3) of $(\hat{x}, \hat{y})$. For $f_2 > 0$ we find that $\frac{(I_2 - f_2 - bI_1)}{(bI_1 - I_2)}$ has to be negative for $(\hat{x}, \hat{y})$ to have pure imaginary eigenvalues (complex conjugated eigenvalues with real part equals 0). In this case the inner fixed point $(\hat{x}, \hat{y})$ may be either a centre, a stable, or an unstable spiral, with further analysis needed to determine the stability.
