# Supplementary material for: Studying mixed-species biofilms of Candida albicans and Staphylococcus aureus using evolutionary game theory
Source: PLoS One. 2024 Mar 6;19(3):e0297307. doi: 10.1371/journal.pone.0297307 (PMC10917284; doi:10.1371/journal.pone.0297307)
Supplement: S2 File — (PDF) [file pone.0297307.s002.pdf]

Studying mixed-species biofilms of *Candida albicans* and *Staphylococcus aureus* using Evolutionary Game Theory

- Supporting Information 1 -

Sybille Dühring[1*], Stefan Schuster[1],

1 Dept. of Bioinformatics, Friedrich-Schiller-University Jena, Jena, Germany

* sybille.duehring@uni-jena.de

The dynamical behaviour of the game - simulation results of the case $I_2 = 0$

Fig 1 shows possible population profiles of the game over time under varying parameter conditions given in Table 1. For the simulations, we assumed no interference from third parties and set the artificial farnesol level f_{ar} equal to zero. As morphology-dependent parameters, we fix u_Y, u_H, u_S, and σ arbitrarily and assume a variation of E_Y, E_H, and E_S to be due to a variation in the available nutrients of the environment n. For detailed information see the main manuscript.

Depending on the parameter values, we see different outcomes for the strategy fractions of *C. albicans* and *S. aureus*. Fig 1 shows the simulation results of the case $I_2 = 0$. It can be seen in Fig 1a and b as well as Fig 1d and e that the course of the curve and the time needed to reach a fixed point are parameter-dependent. The simulations of the case $I_2 \neq 0$, given in the main manuscript, are very similar reaching the same fixed points. This is the case as the dynamical behaviour of the curves of both cases is qualitatively the same, but reached with different parameter values.

Table 1. Parameter values corresponding to the different cases shown in Figure 1.

Parameter	Case $(0,0)$ (Fig. 1a/Fig. 1b)	Case $(1,0)$ (Fig. 1c)	Case $(0,1)$ (Fig. 1d/Fig. 1e)	Case $(1,1)$ (Fig. 1f)
n	0.3 / 1	1.5	0.83 / 1.5	1.5
I_1	0.2 / 1	0.2	0.2	0.1
I_2	0	0	0	0
f_2	-0.5	-0.05	-0.05 / 0.05	0.05

For all cases: $u_Y = 2$, $u_H = 1$, $u_S = 2.5$, $\sigma = 3$, $f_1 = 0.02$, $r = 1.5$, $b = 0.4$, $f_{ar} = 0$ with all values listed having arbitrary units.

The influence of artificially added farnesol (f_{ar}) and nutritional exploitation by *S. aureus* (I_2) on the system - additional simulation results of the case $I_2 \neq 0$ and the case $I_2 = 0$

To study the influence of nutrients (n) and the change in growth caused by farnesol (f_{ar} and f_2) on the mixed-species biofilm, we calculate the strategy fractions x and y at time

step 100 (a.u.), where a quasi-equilibrium has already been established. We set $I_2 = 0.12$ to simulate the case of exploitation or $I_2 = 0$ in case of no exploitation. The parameter values of $u_Y, u_H, u_S, \sigma, f_1$, r, and b are given in Table 1. We assume that $I_1 = 0.2$ and simulate over $n \in [0, 5]$, $f_{ar} \in [0, 0.1]$ and $f_2 \in [-0.1, 0.1]$. The results are shown for selected f_2 levels in Fig 2 (with $I_2 \neq 0$) and Fig 3 (with $I_2 = 0$).

We find different population profiles of *C. albicans* and *S. aureus* depending on the number of nutrients (n), the levels of change in growth caused by farnesol (f_{ar} and f_2) and the gain and loss resulting from the exploitation (I_2). We distinguish between the two cases of exploitation ($I_2 \neq 0$) and no exploitation ($I_2 = 0$). For each case we distinguish between different degrees of severity (beneficial, unsusceptible, low, moderate and high levels of change) in which released farnesol (f_2) is affecting the *S. aureus* population. We further distinguish between different levels of change (high and low) caused by artificially added farnesol (f_{ar}). For detailed information see the main manuscript.

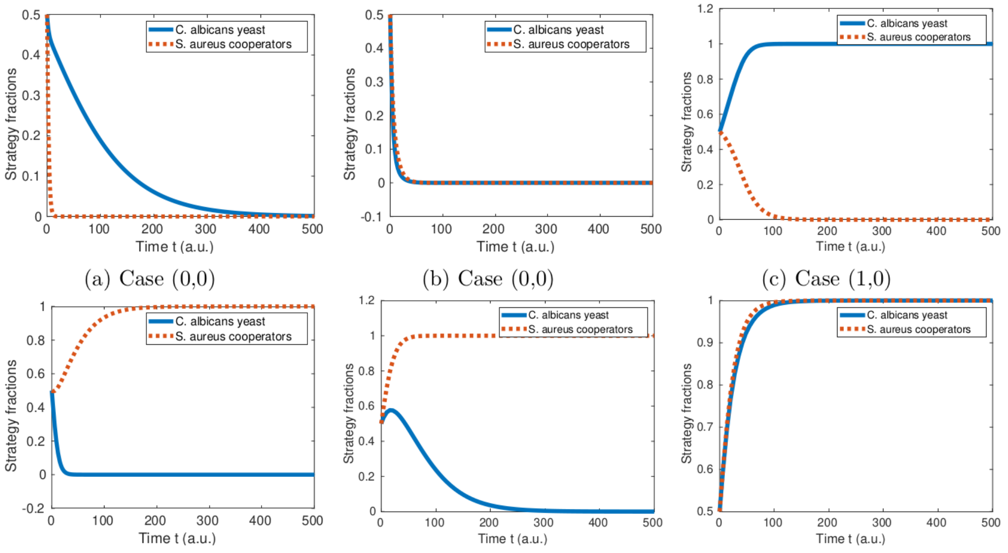

(a) Case (0,0) (b) Case (0,0) (c) Case (1,0)

(d) Case (0,1) (e) Case (0,1) (f) Case (1,1)

Fig 1. The dynamical behaviour of the game. The simulations show possible population profiles of *C. albicans* and *S. aureus* over time for varying parameter settings. For the parameter details of the simulations see Table 1. All six simulations start with a 50/50 mix of hyphae and yeast cells in the *C. albicans* population and a 50/50 mix of exploiting and cooperating cells in the *S. aureus* population. Observations at the end of the simulations: a) and b) All *C. albicans* cells adopt the hyphae strategy and all *S. aureus* cells adopt the exploitation strategy. c) All *C. albicans* cells adopt the yeast strategy and all *S. aureus* cells adopt the exploitation strategy. d) and e) All *C. albicans* cells adopt the hyphae strategy and all *S. aureus* cells adopt the cooperation strategy. f) All *C. albicans* cells adopt the hyphae strategy and all *S. aureus* cells adopt the cooperation strategy. The simulations 1a and b as well as 1d and e end in the same biofilm state. However, the course of the curve and the time needed to reach a fixed point is parameter-dependent.

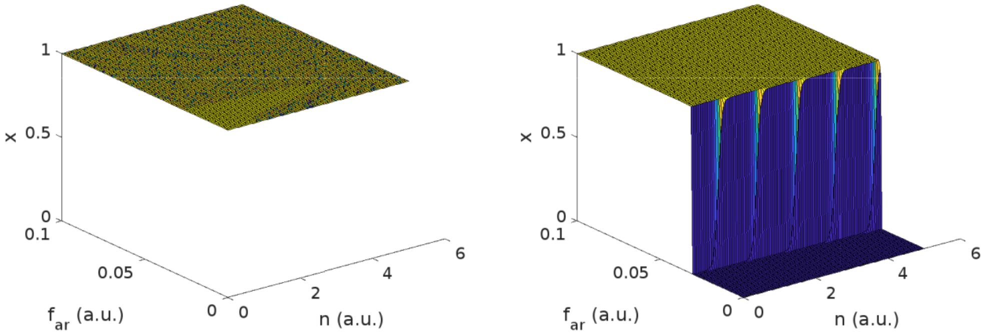

(a) Farnesol $f_2 < 0$ (b) Farnesol $f_2 > 0.06$

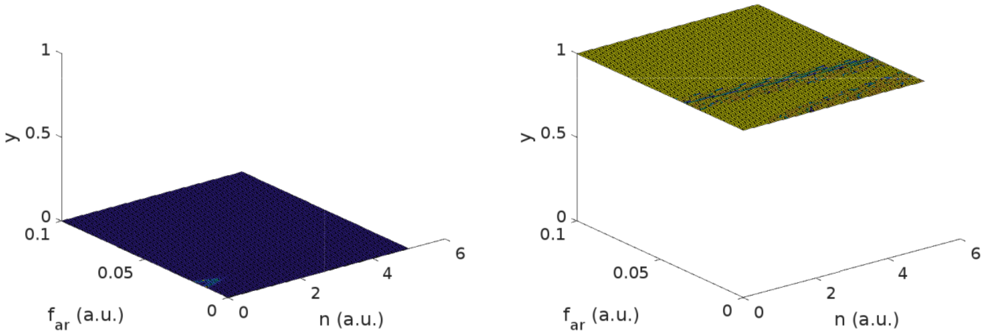

(c) Farnesol $f_2 < 0$ (d) Farnesol $f_2 > 0.06$

Fig 2. Simulation results of the case $I_2 \neq 0$. For selected f_2 values the strategy fractions of *C. albicans* (x) and *S. aureus* (y) are depicted at time step 100 (a.u.) with varying nutrient levels (n) and levels of growth change caused by farnesol (f_{ar} and f_2). Fig 2a and b show selected findings of *C. albicans* while Fig 2c and d show selected findings of *S. aureus*. The population profiles of *C. albicans* and *S. aureus* differ depending on the number of nutrients (n), the levels of change in growth caused by farnesol (f_{ar} and f_2) and the gain and loss resulting from the exploitation (I_2; see also Fig 3 for the results of the case $I_2 = 0$ for comparison).

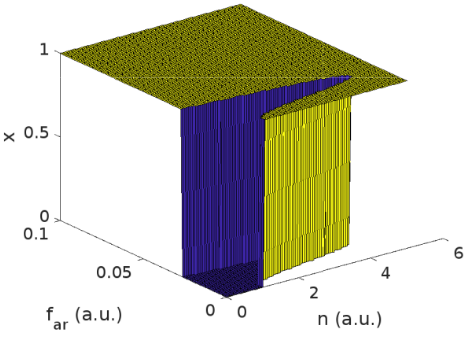

(a) Farnesol $f_2 = -0.04$

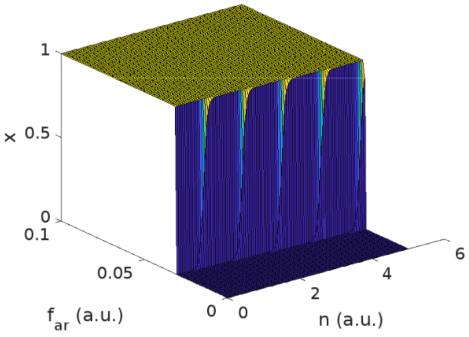

(b) Farnesol $f_2 > 0$

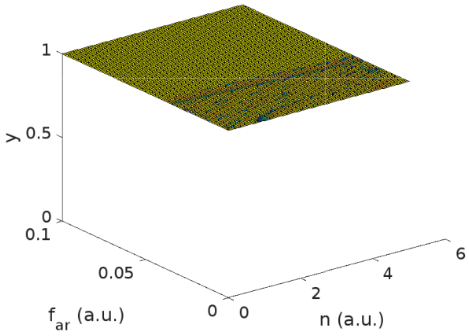

(c) Farnesol $f_2 > 0$

Fig 3. Simulation results of the case $I_2 = 0$. For selected f_2 values the strategy fractions of *C. albicans* (x) and *S. aureus* (y) are depicted at time step 100 (a.u.) with varying nutrient levels (n) and levels of growth change caused by farnesol (f_{ar} and f_2). Fig 3a and b show selected findings of *C. albicans* while Fig 3c shows a selected finding of *S. aureus*. The population profiles of *C. albicans* and *S. aureus* differ depending on the number of nutrients (n), the levels of change in growth caused by farnesol (f_{ar} and f_2) and the gain and loss resulting from the exploitation (I_2; see also Fig 2 for the results of the case $I_2 \neq 0$ for comparison).
